# Impact of Pollution on Mental Health: A Systematic Review of Associations, Methodological Challenges, and Future Directions

**DOI:** 10.1002/hsr2.72514

**Published:** 2026-05-13

**Authors:** Sreeni Chadalavada, Alen Shahini, Yuki Hagiwara, Massimo Salvi, Ekta Sharma, Sonja March, Tracy Kolbe‐Alexanders, Ravinesh Deo, Aly Farag, Prabal Datta Barua, Filippo Molinari, U. Rajendra Acharya

**Affiliations:** ^1^ School of Engineering University of Southern Queensland Springfield Queensland Australia; ^2^ Biolab, PolitoBIOMedLab, Department of Electronics and Telecommunications Politecnico di Torino Turin Italy; ^3^ Fraunhofer Institute for Cognitive Systems IKS Munich Germany; ^4^ School of Mathematics, Physics and Computing University of Southern Queensland Springfield Queensland Australia; ^5^ School of Psychology and Wellbeing, the Centre for Health Research, and the Manna Institute of University of Southern Queensland Ipswich Springfield Queensland Australia; ^6^ School of Health and Medical Sciences, the Centre for Health Research, and the Manna Institute of University of Southern Queensland Ipswich Springfield Australia; ^7^ Computer Vision and Image Processing Laboratory, Department of Electrical and Computer Engineering University of Louisville Louisville Kentucky USA; ^8^ School of Business (Information System) University of Southern Queensland Springfield Queensland Australia; ^9^ Centre for Health Research University of Southern Queensland Springfield Queensland Australia

**Keywords:** artificial intelligence, cognitive function, machine learning, mental health, pollution, public health

## Abstract

**Background and Aim:**

Environmental pollutants, including contaminated air, harmful chemicals, and excessive noise, are increasingly prevalent in modern society. These contaminants can significantly affect mental well‐being, a fundamental determinant of cognitive functioning, emotional regulation, interpersonal relationships, life satisfaction, and overall physical health.

**Methods:**

This systematic review examines the effects of various types of pollution, such as air pollution, noise pollution, and chemical contaminants, as well as their interactions, on mental health outcomes across diverse populations.

**Results:**

Our search identified 61 high‐quality studies that met our inclusion criteria. Among quantitative studies, 81% (*n* = 49) reported a significant association between pollution and mental health outcomes. Air pollution was the most frequently studied factor, with 50% confirming an association. In contrast, chemical pollution showed the lowest positive associations, with only 10% reporting an association. Our analysis reveals critical limitations in current research, particularly regarding data availability and quality, with most mental health data sets being limited in temporal scope and geographical coverage. While we discuss Artificial Intelligence as a prospective methodological framework to improve the precision and efficiency of future studies, we emphasize that its effective implementation fundamentally depends on addressing underlying data limitations. Specifically, spatiotemporal models can address exposure misclassification, attention mechanisms can handle confounding complexity, and deep learning can manage temporal variability, but all require systematic improvements in data collection infrastructure.

**Conclusion:**

This review highlights the urgent need for standardized mental health monitoring systems, interdisciplinary collaboration, and the development of comprehensive data collection frameworks as essential prerequisites for leveraging advanced analytical methods in understanding pollution–mental health relationships.

## 1 Introduction

Environmental pollution, including air, chemical, and noise pollution, is increasingly pervasive in modern societies and represents a growing threat to human well‐being [1, 2]. Beyond its established physical health effects, pollution has been recognized as a major environmental stressor with significant implications for mental health. Mental well‐being is essential for cognitive functioning, emotional regulation, and social interactions, and is therefore a critical public health concern [3].

Among environmental stressors, air, chemical, and noise pollution are particularly relevant to mental health because of their widespread distribution and potential for chronic exposure across large populations [2]. Air pollution arises from anthropogenic sources such as transportation, industrial activities, household energy use, and agriculture, as well as natural sources including wildfires and dust events. Commonly studied air pollutants include particulate matter (PM₂.₅, PM₁₀), nitrogen dioxide (NO₂), sulfur dioxide (SO₂), carbon monoxide (CO), ozone (O₃), and volatile organic compounds (VOCs), which have been associated with adverse mental health outcomes across different populations [4]. Chemical pollution originates from industrial processes, agricultural practices, waste disposal, and accidental releases, leading to exposure to contaminants such as heavy metals, polycyclic aromatic hydrocarbons (PAHs), per‐ and polyfluoroalkyl substances (PFAS), polychlorinated biphenyls (PCBs), and dioxins through air, water, food chains, and soil [3, 5, 6, 7]. Noise pollution, primarily driven by transportation systems, industrial activity, construction, and urbanization, contributes to annoyance, stress, sleep disturbance, and cognitive impairment, with increasing evidence linking chronic noise exposure to adverse mental health outcomes [8].

Despite the growing body of evidence linking environmental pollution to mental health, substantial gaps remain in current research [4]. Many studies focus on individual pollutants in isolation, rely on heterogeneous exposure assessment methods, or are constrained by limited temporal and geographical coverage. These limitations hinder the ability to capture real‐world exposure scenarios characterized by spatial–temporal variability, co‐exposure to multiple pollutants, and complex interactions with socioeconomic, behavioral, and health‐related factors. As a result, conventional statistical approaches often struggle to model non‐linear relationships adequately and to control for multiple confounders when investigating pollution–mental health associations.

Accordingly, this paper presents a systematic review of the literature on the relationship between environmental pollution and mental health, conducted in accordance with PRISMA guidelines. The main contributions of this work are as follows:

– a comprehensive synthesis of evidence linking air, chemical, and noise pollution to mental health outcomes, based on 61 high‐quality studies published between 2016 and 2024;
– a critical assessment of methodological approaches used in existing studies, highlighting key limitations in exposure assessment, data quality, and confounding control;
– a discussion of how artificial intelligence (AI)‐based methods could address these research challenges in future studies, enabling more robust, scalable, and integrative analyses of pollution–mental health relationships.

In recent years, AI methods have shown potential for advancing environmental mental health research. AI‐based methods can integrate heterogeneous data sources, including environmental monitoring data, health records, and socioeconomic indicators. They can also model complex spatial–temporal patterns and non‐linear interactions that are difficult to capture using traditional analytical approaches [9, 10, 11]. However, the effective application of AI in this domain critically depends on the availability, quality, and standardization of pollution and mental health data. These issues are systematically identified and discussed throughout this review.

### 1.1 Related Reviews

**Figure 1:**
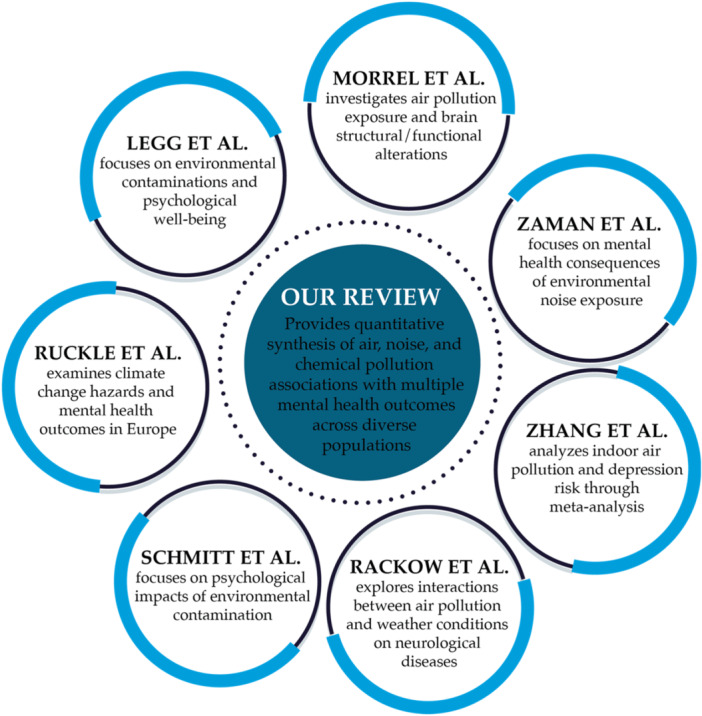
Comparison of our review paper with existing literature reviews.

Several literature reviews have investigated the relationship between environmental pollution and mental health (Figure 1), but important limitations remain.

Single‐pollutant reviews have provided foundational evidence but with narrow scope. Schmitt et al. [12] focused on chronic contamination scenarios, excluding acute exposures and emerging pollutants, and did not consider modern analytical methods or real‐time monitoring. Zaman et al. [8] examined the mental health consequences of noise pollution, but their review was limited to a single pollutant and did not address methodological challenges related to exposure assessment or data integration. Legg et al. [13] analyzed mental health impacts in industrial contamination contexts, overlooking other major environmental pollutants and the potential of advanced computational approaches.

Recent multi‐domain reviews have addressed broader aspects of environmental exposures, but each has important limitations. Rückle et al. [14] conducted a comprehensive systematic review on climate change hazards and mental health in Europe, identifying multiple pathways linking air pollution, floods, wildfires, and temperature extremes to depression, anxiety, and cognitive impairment. However, their review acknowledged extreme heterogeneity in outcome measurements and study designs, making cross‐hazard comparisons difficult and preventing meta‐analytic synthesis. Rackow et al. [15] systematically reviewed bidirectional interactions between air pollution and weather conditions on mortality and mental/neurological diseases. They found synergistic effects between combined exposures and adverse health outcomes. However, the review was geographically limited to Europe and North America, and substantial heterogeneity in study designs and exposure definitions precluded meta‐analysis.

Morrel et al. [16] conducted a systematic review of MRI studies examining air pollution and brain structure/function, identifying associations between pollutants (particularly PM₂.₅) and structural and functional brain alterations. However, their review focused exclusively on neuroimaging outcomes and was limited to Western countries with relatively low pollution levels.

Zhang et al. [17] conducted a systematic review and meta‐analysis examining associations between indoor air pollution (from solid fuel use and secondhand smoke) and depression risk. They found increased depression risk overall, with stronger associations for solid fuel use than secondhand smoke. However, studies were predominantly from China and the USA, exposure was assessed as binary (precluding dose‐response analyses), and relied primarily on self‐report questionnaires.

These reviews have important limitations. Each examined only one pollution type (air pollutants, climate hazards, weather‐pollution interactions, or indoor pollution), focused on specific outcomes (neuroimaging, clinical symptoms, or specific diseases), examined specific settings (outdoor or indoor), or covered limited geographic regions.

In contrast to these previous works, the present review adopts a comprehensive perspective by jointly examining air, noise, and chemical pollution and their associations with mental health outcomes. By considering interactions among pollutants and discussing advanced computational approaches to support data integration and real‐time analysis, this study provides a more holistic framework for understanding pollution‐related mental health risks and informing evidence‐based policies and interventions.

### 1.2 Research Questions

This review aims to address the following research questions:

– What is the current evidence of associations between different types of environmental pollution (air, water, noise, light, etc.) and specific mental health outcomes?
– What methodological approaches have been employed to study these associations, and what are their strengths and limitations?
– How can artificial intelligence techniques enhance research on the relationship between environmental pollution and mental health?
– What are the key ethical considerations and challenges in applying AI to environmental mental health research?
– What future research directions should be prioritized to advance understanding of environmental pollution impacts on mental health using AI approaches?

## 2 Article Search and Selection Methods

This systematic review was conducted in accordance with the PRISMA (Preferred Reporting Items for Systematic Reviews and Meta‐Analysis) guidelines, in line with the EQUATOR Network recommendations [18]. In June 2024, a comprehensive literature search was performed in PubMed, Scopus, and Web of Science using Boolean combinations of the keywords “Environmental”, “Air”, “Noise”, “Pollution”, “Well‐being”, “Mental Health”, and “Contaminants”. The final search string was: (“Environmental” OR “Air” OR “Noise” OR “Pollution”) AND (“Well‐being” OR “Mental Health” OR “Contaminants”). These databases were selected for their broad coverage of peer‐reviewed, high‐quality scientific literature. Articles published between 2016 and June 2024 were included.

The initial search identified 421 records. After removing 213 duplicates, 208 unique articles were screened based on title and abstract. Studies were excluded if they were not peer‐reviewed, not published in English, not focused on human populations, not directly relevant to associations or causal relationships between pollution and mental health, or not available in full text. Non‐English publications were excluded to ensure consistency, methodological rigor, and formal peer review, acknowledging that this may introduce some limitations.

Following this screening step, 115 articles were excluded. The remaining 93 studies underwent full‐text assessment, including evaluation of quality and relevance. Study classification and categorization, including quality assessment, were performed independently by two co‐authors (A.S. and Y.H.).

### 2.1 Quality Assessment Protocol

Each of the 93 full‐text articles was independently evaluated by both reviewers using a standardized quality assessment framework specifically adapted for environmental health research (detailed criteria in Appendix A). The assessment evaluated five key methodological dimensions:

1. Study design quality (0–3 points): Appropriateness and rigor of study design (e.g., longitudinal vs. cross‐sectional); clarity of exposure and outcome definitions; establishment of temporal relationships; sample size adequacy and statistical power considerations.
2. Exposure assessment (0–3 points): Validity and reliability of pollution measurement methods (e.g., monitoring stations, satellite data, personal sensors); spatial and temporal resolution of exposure data; consideration of relevant exposure windows; assessment of exposure misclassification.
3. Mental health assessment (0–3 points): Use of validated mental health instruments; diagnostic rigor and clinical relevance; specificity of mental health outcomes; assessment of measurement validity in the study population.
4. Statistical analysis (0–3 points): Appropriateness of analytical methods for research question; sensitivity analyses to test robustness of findings; transparency in handling missing data.
5. Reporting quality (0–3 points): Completeness and transparency of methodological reporting following relevant guidelines (e.g., STROBE); clarity in presentation of results; discussion of limitations; reproducibility potential.

For primary empirical studies, the framework assessed these five dimensions as described above. For systematic reviews and meta‐analyses, adapted criteria were applied focusing on: (1) comprehensiveness and transparency of search strategy; (2) critical appraisal and quality assessment of included studies; (3) appropriateness of synthesis methods (meta‐analytic or narrative); (4) nuanced interpretation of evidence accounting for heterogeneity; and (5) reporting completeness following PRISMA or similar guidelines (see Appendix A for detailed adapted criteria).

Studies could achieve a maximum score of 15 points across all five dimensions. Articles were included if they achieved a minimum score of 7 points. This threshold was selected to ensure methodological quality while maintaining inclusiveness appropriate for this interdisciplinary field. Studies with fundamental design flaws or insufficient reporting were excluded. Discrepancies in scoring were resolved through discussion between the two reviewers until consensus was reached. In cases where consensus could not be initially reached (*n* = 4 studies), a third reviewer (M.S.) was consulted.

**Figure 2:**
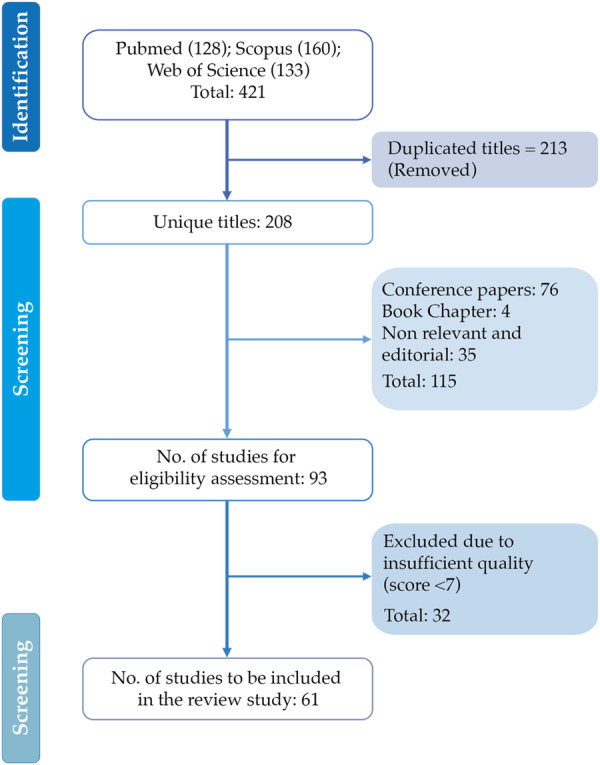
Selection process for relevant studies following PRISMA criteria. The total number of included studies is 61. Exclusions at each stage are also indicated, with the number and type of articles excluded next to the flowchart.

Following quality assessment, 32 studies were excluded due to insufficient methodological quality, resulting in 61 high‐quality studies for final inclusion: 52 primary empirical studies and 9 systematic reviews/meta‐analyses. Figure 2 presents the PRISMA flow diagram summarizing the identification, screening, eligibility assessment, and final inclusion of studies. Detailed quality assessment scores for each included study are reported in the Supporting Information S1: Tables S1–S4.

## 3 Results

Among the 61 reviewed articles, 34 focused on air pollution, 8 on chemical pollution, 3 on noise pollution, 2 on air and noise pollution, and 14 on combinations of multiple types of pollution. The predominant research methodology used in these studies was observational, although a smaller number of experimental studies were also included.

### 3.1 Air Pollution

Supporting Information S1: Table S1 summarizes studies on the impact of air pollution on mental health (*n* = 36). The collective evidence from numerous studies suggests a robust association between exposure to air pollutants and various adverse mental health outcomes.

Research consistently demonstrates that long‐term exposure to air pollution, particularly particulate matter (PM_2.5_ and PM_10_) and gaseous pollutants (NO_2_, SO_2_, CO, and O_3_), is associated with increased risks of depression, anxiety, and other psychiatric disorders [19, 20, 21]. These associations have been observed in various demographic groups, including children [22, 23], adolescents [24, 25], adults [26, 27, 28, 29, 30], and older populations [21, 31, 32]. The impact of air pollution on mental health appears to be moderated by various factors. Socioeconomic status, gender, and pre‐existing health conditions have been identified as significant modifiers of the relationship between air pollution and mental health outcomes [33, 34, 35, 36]. For instance, studies have found that women, individuals with lower education and income levels, and those with pre‐existing health conditions may be more susceptible to the mental health effects of air pollution [29, 37].

Several studies have explored the potential mechanisms through which air pollution may affect mental health. These include increased inflammation, oxidative stress, and alterations in neurotransmitter systems [38, 39, 40, 41]. Additionally, research has suggested that air pollution may indirectly impact mental health through its effects on physical health, sleep quality, and overall quality of life [42, 43, 44]. The relationship between air pollution and mental health extends beyond individual‐level effects. Studies have found associations between air pollution levels and increased utilization of mental health services, including outpatient visits, emergency department trips, and hospitalizations [9, 45]. This highlights the broader public health implications of air pollution on mental health care systems.

Recent research has also examined the impacts of air pollution on mental health in the context of specific events or populations [46]. These include studies on maternal exposure during pregnancy and subsequent child mental health [47], the effects of air pollution on cancer survivors' mental health [48], and the impact of air pollution on mental health in the context of the COVID‐19 pandemic [49, 50].

The cumulative evidence strongly supports a significant association between air pollution exposure and adverse mental health outcomes. This relationship is observed across various pollutants, geographical regions, and demographic groups, underscoring the global nature of this environmental health challenge.

### 3.2 Chemical Pollution

Supporting Information S1: Table S2 presents studies (*n* = 8) exploring the relationship between different types of chemical pollution and mental health indicators.

Studies have highlighted the vulnerability of children to the mental health impacts of chemical pollution. Maternal exposure to polycyclic aromatic hydrocarbons during pregnancy has been associated with poorer mental health outcomes in children [51]. In adults, research has shown associations between exposure to heavy metals in topsoil and increased risk of mental disorders [52]. Communities affected by chemical contamination often experience significant psychosocial stress. Studies on per‐ and polyfluoroalkyl substances contamination have revealed heightened anxiety, uncertainty, and distrust among affected residents [53, 54]. Indigenous communities exposed to industrial pollutants, such as methylmercury from gold mining, also face unique mental health challenges [55]. The interplay between chemical pollutants and other factors, such as alcohol consumption, can exacerbate mental health risks [56]. Despite the growing evidence, significant knowledge gaps remain in understanding the full extent of chemical pollution's impact on mental health [12, 13].

The evidence suggests that exposure to chemical pollutants can increase the risk of mental health disorders and compromise psychological well‐being.

### 3.3 Noise Pollution

Supporting Information S1: Table S3 presents studies (*n* = 5) on noise pollution and its impact on mental health across different populations and contexts.

A large‐scale study in Germany found that individuals experiencing high noise annoyance had double the risk of impaired mental health, with women being more affected than men [57]. Similar findings were reported in China, where higher noise pollution exposure was significantly associated with worse mental health outcomes [58].

The impact of noise pollution on mental health appears to be particularly pronounced in urban environments [59]. Studies have identified traffic and construction as primary sources of noise that negatively affect mental well‐being [58]. Moreover, long‐term exposure to noise pollution during youth and adolescence has been associated with increased anxiety levels later in life [25, 60]. While the link between noise pollution and anxiety seems robust, its association with other mental health conditions, such as depression and psychosis, is less clear. Some studies have found no significant relationship between noise pollution and these disorders [61]. The effects of noise pollution on mental health may also interact with other environmental factors. For instance, when considered alongside air pollution and green space availability, the impact of noise pollution on mental health outcomes can vary [61].

This evidence strongly suggests that noise pollution is a significant environmental stressor that can negatively impact mental health.

### 3.4 Combined Pollution and Miscellaneous Studies

Supporting Information S1: Table S4 summarizes papers (*n* = 14) examining the combined effects of multiple pollutants, as well as other environmental factors, to provide a more comprehensive understanding of the complex relationship between environmental pollution and mental health.

Studies investigating the joint impact of air and noise pollution have revealed that both pollutants can contribute to poor mental health outcomes, albeit through different pathways [62]. For instance, Dzhambov et al. [63] found that daytime noise directly impacted mental health, while air pollution had indirect effects. Similarly, research using UK Biobank data uncovered associations of exposure to both PM2.5 and road traffic noise with various mental health issues [64].

The interplay between different environmental factors, including green spaces, air pollution, and traffic noise, has been shown to have complex associations with mental health outcomes [61]. Water pollution has also been linked to significant mental health consequences. The Flint water crisis, for example, led to heightened stress, anxiety, depression, and distrust among affected residents [65]. Studies on wildfire smoke exposure have indicated potential impacts on mental health, particularly during prolonged events, although the evidence remains somewhat inconsistent [1, 66].

Research has also pointed to the potential role of environmental pollutants in triggering more severe mental health conditions, such as schizophrenia [67]. Additionally, the impact of pollution on mental health may be moderated by factors such as socioeconomic status, pre‐existing health conditions, and individual perceptions of environmental risks [68, 69]. Recent research has employed advanced techniques, such as data mining and structural equation modeling, to better understand the complex relationships between various environmental factors and mental health [70, 71].

The evidence from combined pollution and miscellaneous studies emphasizes the complex, interconnected nature of environmental pollutants and their impacts on mental health.

## 4 Discussion

### 4.1 Main Findings

**Figure 3:**
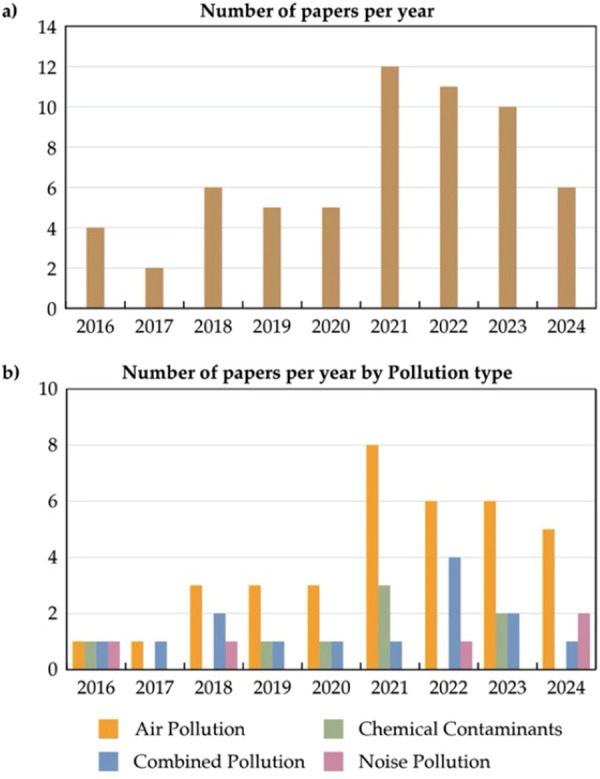
Temporal distribution of the reviewed papers, categorized by (a) year and (b) pollution type.

Publication trends show growing research interest in the relationship between pollution and mental health (Figure 3a). Publications increased steadily after 2018, peaked in 2021, and remained high over the years. Air pollution was the most studied exposure, followed by chemical pollution, whereas noise pollution was less commonly examined (Figure 3b). Since 2021, more studies have examined combined or multi‐pollutant exposures, highlighting a shift toward more integrative approaches.

**Figure 4:**
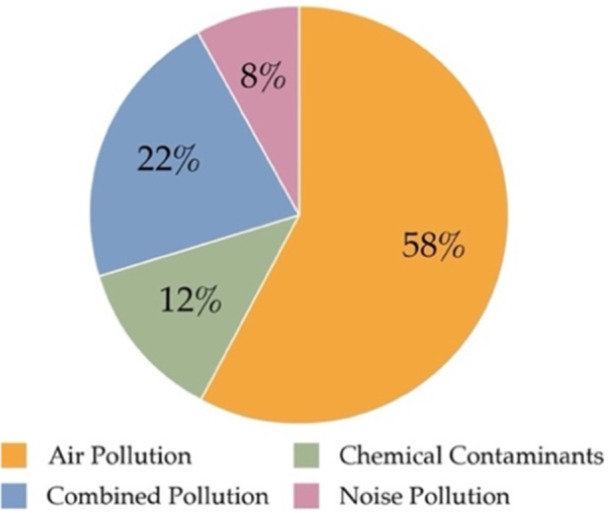
Distribution of papers by pollution type.

The distribution of studies by pollution type (Figure 4) shows that more than half examined air pollution (*n* = 36), while fewer studies focused on chemical (*n* = 8) and noise pollution (*n* = 5). A substantial number of studies (*n* = 14) addressed multiple pollutants, reflecting growing interest in interaction effects.

**Figure 5:**
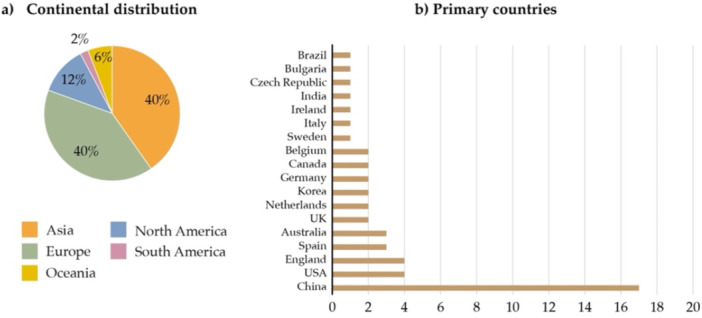
Geographical distribution of studies on pollution and mental health.

Geographically, the literature is unevenly distributed (Figure 5). Most studies focused on Asia and Europe, with China being the most represented country. In contrast, North and South America, Oceania, and other regions remain underrepresented.

**Figure 6:**
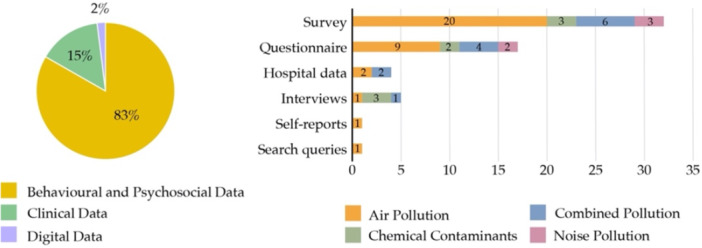
Distribution of papers by personal data type.

Methodologically, most studies used quantitative, longitudinal designs and relied on large‐scale personal data sources, including surveys, questionnaires, hospital records, and self‐reported measures (Figure 6). Behavioral and psychosocial data were used in the majority of studies (83%), while clinical data, such as electronic health records, were less frequently employed. Most studies relied on subjective mental health assessments, with limited integration of objective clinical or digital data sources.

As summarized in Table 1, more than 80% of the quantitative studies reported significant associations between pollution exposure and adverse mental health outcomes across all pollution categories.

**Table 1:** Distribution of studies investigating the association and causality between pollution exposure and mental health.

			Air pollution	Chemical pollution	Combined pollution	Noise pollution	Total
Quantitative (*n* = 52)	Association	Yes	26 (50%)	5 (10%)	8 (15%)	3 (6%)	42 (81%)
		No	6 (11%)	0 (0%)	3 (6%)	1 (2%)	10 (19%)
	Causality	Yes	10 (19%)	3 (6%)	0 (0%)	0 (0%)	13 (25%)
		No	0 (0%)	0 (0%)	0 (0%)	0 (0%)	0 (0%)
		No evaluation	22 (43%)	2 (4%)	11 (21%)	4 (7%)	39 (75%)
Qualitative (*n* = 15)	Association	Yes	3 (20%)	5 (33%)	4 (27%)	1 (7%)	13 (87%)
		No	1 (6.5%)	0 (0%)	1 (6.5%)	0 (0%)	2 (13%)
	Causality	Yes	2 (13%)	1 (7%)	1 (7%)	0 (0%)	4 (27%)
		No	0 (0%)	1 (7%)	0 (0%)	0 (0%)	1 (7%)
		No evaluation	2 (13%)	3 (20%)	4 (27%)	1 (6%)	10 (66%)

Among the studies that explicitly examined causality (*n* = 13, 25%), all identified evidence suggestive of causal relationships. These studies varied in methodological rigor. Some used observational designs with temporal sequencing or dose–response patterns, while others employed robust causal inference methods (e.g., instrumental variables, propensity score matching, difference‐in‐differences, natural experiments).

The majority of quantitative studies (75%, *n* = 39) did not formally evaluate causality. These studies were limited to association analyses due to cross‐sectional designs, lack of temporal information, or reliance on conventional regression methods that cannot adequately address unmeasured confounding. While advanced causal approaches were occasionally used [40, 47, 48], causal interpretation remains limited by methodological and ethical challenges inherent to environmental health research [64, 69, 72].

**Figure 7:**
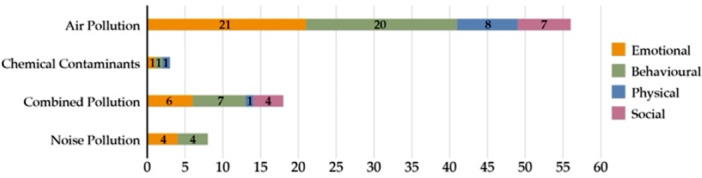
Quantitative studies that investigate mental health impacts across different types of pollution.

**Figure 8:**
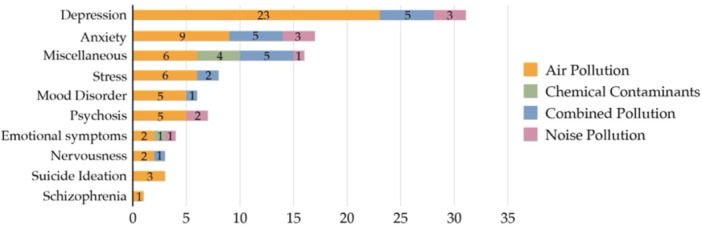
Quantitative studies that found a link between pollution and related mental disorders.

Across studies, we classified pollution‐related mental health impacts into emotional, behavioral, physical, and social domains (Figure 7). Air, noise, and combined pollution most strongly affected emotional and behavioral outcomes, including depression, anxiety, stress, and sleep disturbances, while chemical pollutants were more closely associated with physical and social dimensions of health. Consistent with this pattern, depression and anxiety were the most frequently examined disorders in quantitative studies (Figure 8), while several studies assessed overall mental health without focusing on specific diagnoses.

Findings were consistent across pollutants, populations, and methodologies, strengthening the evidence linking environmental pollution to adverse mental health outcomes (Supporting Information S1: Tables S1–S4).

The frequent overlap between pollution sources has important implications for public health interventions. Industrial activity and traffic often generate multiple stressors simultaneously (e.g., emissions and noise). This suggests that integrated mitigation strategies addressing multiple environmental exposures may be more effective than single‐pollutant approaches, particularly for protecting vulnerable populations (such as low‐income populations, women, older adults, and individuals with pre‐existing health conditions) who are disproportionately affected.

### 4.2 Limitations and Challenges

Several methodological limitations constrain current research on environmental pollution and mental health.

1. Data availability and quality: most studies rely on self‐reported data or retrospective surveys, which are prone to recall and reporting bias. Mental health and pollution data sets are often heterogeneous in temporal resolution, variable definition, and geographical coverage. Moreover, study designs, exposure metrics, and outcome definitions lack standardization, limiting comparability and reproducibility across studies. High‐frequency, longitudinal data at national or regional levels remain scarce, posing a major obstacle to robust causal inference.
2. Complexity of exposure assessment: individuals are typically exposed to multiple pollutants from diverse sources that vary across time and space. Many studies rely on ecological‐level exposure estimates and regression‐based models that fail to capture individual‐level variability or cumulative exposure. This limits the identification of direct causal relationships.
3. Confounding and effect modification: pollution–mental health associations are influenced by numerous interacting factors. These include socioeconomic status, age, gender, pre‐existing mental health conditions, environmental co‐exposures (e.g., noise, temperature, green space), behavioral patterns, and temporal dynamics. Traditional statistical approaches often cannot adequately model complex, non‐linear interactions, leading to residual confounding and biased effect estimates. Geographic differences in pollutant levels, healthcare systems, diagnostic practices, and cultural perceptions of mental health introduce additional heterogeneity.
4. Geographic representation: existing studies are concentrated in Asia, Europe, and North America, with limited representation from Africa, South America, and parts of South Asia. This imbalance restricts the generalizability of current evidence to underrepresented regions and populations.

### 4.3 Future Directions

AI methods could help address the methodological inconsistencies identified in current pollution–mental health research. Future work should prioritize reproducible analytical pipelines that explicitly link current limitations to appropriate AI‐based strategies (Table 2), rather than isolated algorithmic applications. To provide a systematic roadmap for researchers and practitioners, Table 2 presents a comprehensive framework that maps each identified empirical limitation in current pollution–mental health research to specific AI‐based solutions and their expected methodological improvements.

**Table 2:** Mapping of current limitations and the potential role of AI for future pollution and mental health research.

Current limitation	AI‐based solution	Expected improvement
Data availability and quality	Deep learning for data imputation; transfer learning for small datasets	Better handling of missing data; ability to leverage knowledge from data‐rich regions
Methodological variability	Standardized deep learning pipelines; automated feature extraction	More consistent analysis across studies; reduced reliance on manual feature engineering
Complex exposure assessment	Graph neural networks; spatio‐temporal models	Better modeling of pollution dispersion; integration of multiple exposure pathways
Confounding factors	Attention mechanisms; causal AI approaches	Dynamic weighting of relevant factors; better isolation of pollution effects
Geographic representation	Federated learning; domain adaptation	Enable multi‐site studies while preserving privacy; better generalization across regions

A central challenge remains the integration of heterogeneous environmental and mental health data, including air quality [23], noise exposure [25], chemical contamination indicators [52], and longitudinal mental health outcomes. To apply AI methods effectively, future research will need standardized data preprocessing to address missing data [37], spatiotemporal misalignment [25], and inconsistent variable definitions, as well as robust feature extraction strategies [21].

As shown in Table 2, graph‐based and spatiotemporal models are well‐suited to capture complex exposure patterns, while attention‐based and causal AI approaches could help identify dynamically relevant factors across time and population subgroups. However, these methods should complement, not replace, rigorous study design and domain knowledge.

Model evaluation should combine standard performance metrics (e.g., AUC [52], MSE, *R*² [9]) with uncertainty estimation to support risk assessment. AI‐based systems could enable near real‐time monitoring [19], but their reliability depends on standardized, FAIR‐compliant data infrastructures supported by public health institutions [3].

Interpretability and transparency remain essential for scientific credibility and policy relevance. Moreover, climate‐related variables should be systematically incorporated, given their influence on pollutant dynamics. Finally, interdisciplinary collaboration and explicit links between empirical findings and regulatory frameworks are necessary to translate methodological advances into actionable public health insights.

### 4.4 Ethical Considerations

AI applications in environmental mental health research raise ethical challenges requiring dedicated governance frameworks beyond technical considerations. Given the sensitivity of mental health data, strict compliance with data protection regulations such as GDPR and HIPAA is essential [73]. Privacy‐preserving approaches (e.g., differential privacy, federated learning, and secure multi‐party computation) are essential when integrating mental health and environmental exposure data [74]. Combining data sets from environmental monitoring, healthcare systems, and personal sensing technologies requires robust anonymization and secure data infrastructures [75].

Bias and fairness represent additional ethical concerns. AI models trained on unbalanced or incomplete data may reinforce existing disparities in mental health outcomes [76, 77]. This risk is particularly concerning given the strong links between pollution exposure, socioeconomic disadvantage, and structural inequality. Systematic bias assessment, algorithmic auditing, and inclusive data collection can mitigate disproportionate impacts on vulnerable populations [78].

Informed consent poses further challenges in AI‐driven research, particularly for longitudinal studies and adaptive models whose objectives or parameters may evolve over time [79]. Dynamic consent mechanisms that allow participants to update their preferences can enhance transparency and trust, especially in community‐based environmental health research [80].

## 5 Conclusion

Environmental pollution consistently harms mental health. Among the 61 high‐quality studies reviewed, PM_2.5_ and NO_2_ showed the strongest associations with depression, anxiety, and suicide risk. Chemical and noise exposures also showed adverse effects, though evidence remains more limited. Major limitations constrain progress. Exposure assessment varies widely across studies, longitudinal mental health data are scarce, and most evidence comes from Asia, Europe, and North America. Ethical challenges related to data privacy, algorithmic bias, and informed consent require rigorous governance frameworks. Future work should prioritize longitudinal studies with standardized exposure metrics, expand geographic coverage through international collaboration, and develop AI methods with attention to transparency and clinical utility.

## Author Contributions

**Sreeni Chadalavada:** data curation, formal analysis, writing – original draft. **Alen Shahini:** writing – original draft, formal analysis, visualization. **Yuki Hagiwara:** formal analysis, writing – review and editing, methodology. **Massimo Salvi:** data curation, visualization, writing – original draft. **Ekta Sharma:** writing – review and editing, investigation. **Sonja March:** investigation, writing – review and editing. **Tracy Kolbe‐Alexanders:** Writing – review and editing, validation. **Ravinesh Deo:** writing – review and editing, validation. **Aly Farag:** writing – review and editing. **Prabal Datta Barua:** writing – review and editing. **Filippo Molinari:** writing – review and editing, supervision. **U. Rajendra Acharya:** conceptualization, writing – review and editing, supervision.

## Funding

The authors have nothing to report.

## Ethics Statement

Ethical approval was not required for this study, as it is based exclusively on previously published literature and does not involve the collection or analysis of primary human or animal data.

## Conflicts of Interest

The authors declare no conflicts of interest.

## Transparency Statement

The corresponding author, Massimo Salvi, affirms that this manuscript is an honest, accurate, and transparent account of the study being reported; that no important aspects of the study have been omitted; and that any discrepancies from the study as planned (and, if relevant, registered) have been explained.

## Supporting information

Supporting File

## Data Availability

The data that support the findings of this study are available from the corresponding author upon reasonable request.

## Appendix A Study quality assessment framework

Each paper was evaluated against the following criteria using a standardized assessment framework. For primary empirical studies, the five dimensions below were assessed (Table A1). For systematic reviews and meta‐analyses, adapted criteria were applied (see Table A2).

**Table A1:** Quality assessment criteria for primary empirical studies.

Criterion	Excellent (3 points)	Good (2 points)	Basic (1 point)	Insufficient (0 points)
Study design quality	Prospective longitudinal or quasi‐experimental design with clear temporal sequencing; well‐defined exposure and outcome periods; adequate sample size (*n* > 500); minimal loss to follow‐up (< 20%)	Longitudinal design with some temporal information; clear exposure/outcome definitions; adequate sample size (200 < *n* < 500)	Cross‐sectional design with adequate sample (*n* > 200); basic exposure/outcome definitions; some design limitations acknowledged	Small sample (*n* < 200); poorly defined exposure or outcome; major design flaws; or insufficient information to evaluate
Exposure assessment	Direct measurements with high spatial resolution (< 10 km); multiple pollutants assessed; temporal alignment with mental health outcomes; exposure windows justified based on biological plausibility	Validated exposure data from monitoring stations or models; reasonable spatial resolution (10–50 km); consideration of exposure timing; pollutant‐specific measurements	Indirect exposure assessment (e.g., proximity measures, regional averages); limited spatial/temporal resolution (> 50 km); single pollutant focus	Self‐reported exposure only; or unclear measurement methods; or exposure not clearly linked to study population
Mental health assessment	Validated diagnostic instruments (e.g., structured clinical interviews, ICD/DSM criteria); clinically meaningful outcomes; instruments validated in study population; multiple mental health domains assessed	Validated screening tools (e.g., PHQ‐9, GAD‐7, DASS); standardized questionnaires with established psychometric properties; appropriate for study population	Generic mental health measures or adapted instruments; limited validation evidence; single‐item measures from validated scales	Non‐validated measures; unclear outcome definitions; self‐reported diagnosis without verification; or insufficient information
Statistical analysis	Advanced methods appropriate for exposure–outcome relationship (e.g., mixed models, causal inference approaches); sensitivity analyses performed; effect estimates with 95% CI	Appropriate regression models; adjustment for main confounders (age, sex, socioeconomic status); basic sensitivity or subgroup analyses; transparent reporting of assumptions	Basic regression analysis; some analytical limitations acknowledged; minimal sensitivity testing	Inappropriate statistical methods; or insufficient information to evaluate analytical approach
Reporting quality	Complete reporting following STROBE or similar guidelines; all methods fully described and reproducible; clear presentation of results with effect sizes and uncertainty; comprehensive discussion of limitations	Clear methodological reporting with most details; results well‐presented with appropriate statistics; limitations discussed; study mostly reproducible	Basic reporting of methods and results; some missing details; limited discussion of limitations; partial reproducibility	Incomplete methods; unclear results presentation; limitations not addressed; insufficient information for replication

**Table A2:** Adapted quality assessment criteria for systematic reviews and meta‐analyses.

Criterion	Excellent (3 points)	Good (2 points)	Basic (1 point)	Insufficient (0 points)
Search strategy and study selection	Comprehensive systematic search of multiple databases (≥ 3); PRISMA compliance; search terms and full search strategy reported; duplicate screening process	Systematic search of major databases (2–3); clear inclusion/exclusion criteria; most PRISMA elements present; search terms provided	Single database or limited search; basic selection criteria described; some PRISMA elements	Unclear search strategy; selection criteria not explicitly reported; non‐systematic approach
Quality assessment of included studies	Critical analysis of all included studies; quality scores influence synthesis and conclusions; risk of bias assessed	Quality assessment conducted for most studies; validated tool used; limitations of included studies acknowledged	Minimal quality assessment; studies described but not systematically evaluated using validated tools	No quality assessment of included studies; quality/bias not addressed
Synthesis method	Quantitative meta‐analysis with appropriate statistical methods and heterogeneity assessment, or comprehensive narrative synthesis with a clear conceptual framework	Systematic narrative synthesis; patterns identified across studies; some quantitative summary (e.g., vote counting); heterogeneity discussed	Basic structured summary of findings; limited integration across studies	Simple listing of study results without synthesis or pattern identification
Statistical analysis	Nuanced interpretation accounting for study quality and heterogeneity; clinical/policy implications considered	Clear interpretation with explicit acknowledgment of limitations; evidence gaps identified; reasonable conclusions drawn from available data	Basic interpretation of pooled findings; some limitations mentioned	Overgeneralization beyond available evidence; quality and heterogeneity not considered in interpretation
Reporting quality	Complete reporting following PRISMA, or similar guidelines; all selection decisions documented; data extraction transparent; reproducible search and methods; protocol pre‐registered	Clear methods section; most selection and extraction details reported; largely reproducible; adherence to most reporting guidelines	Basic reporting of methods; some missing details (e.g., excluded studies not listed); partial reproducibility	Incomplete reporting; selection process unclear; not reproducible; major guideline elements missing

Papers scoring below 7 points were excluded to maintain high‐quality standards across the review. This quality assessment framework ensured that only methodologically sound and clinically relevant studies were included in our review, maintaining high standards across all analyzed papers.
