# Supplementary material for: Impact of Pollution on Mental Health: A Systematic Review of Associations, Methodological Challenges, and Future Directions
Source: Health Sci Rep. 2026 May 13;9(5):e72514. doi: 10.1002/hsr2.72514 (PMC13172299; doi:10.1002/hsr2.72514)
Supplement: Supplementary file 1 — Supporting File [file HSR2-9-e72514-s001.docx]

**Supplementary Materials**

**Table S1.** Effect of air pollution on mental health (n=36).

| **Author** | **Study quality and aim** | **Sample size** | **Country** | **Personal Data** | **Pollution Data** | **Method** | **Analytic approach** | **Disorders** | **Findings/ Results** | **Association, Causality** | **Pollution outcomes** |
| --- | --- | --- | --- | --- | --- | --- | --- | --- | --- | --- | --- |
| Dales and Cakmak et al. [22], 2016 | Score 10/15. Cross-sectional analysis to assess mental health influence on the physiologic effects of air pollution. | 1,883 children aged 6-17 | Canada | Physiological measures, Questionnaire | PM 2.5, NO2, O3 data from National AP monitor closest to individual’s neighborhood | Quantitative | Linear regression | MDs, unfavorable emotional symptoms | AP negatively impacts MH; elevated ozone levels increase blood pressure and decrease pulmonary function in children with MDs and those experiencing adverse emotional symptoms. | Yes, - | Emotional, Behavioral |
| Oudin et al. [19], 2017 | Score 12/15. To examine the relationship between AP exposure and asthma, and how this relationship is influenced by MH status and socio-economic factors. | Large dataset of all individuals under 18 | Sweden | Hospital data | Land Use Regression modeling to assess the urban component of NO2 concentrations | Quantitative | Logistic regression | Miscellaneous | Initial hypothesis of MH and education as modifiers not supported weak suggestion of stronger link in children of higher education parents. | No, - | Physical, Social |
| Giovanis et al. [27], 2018 | Score 12/15. To evaluate the effects of air pollution on the health conditions of retired individuals. | 40,000+ individuals aged 50+ | Europe | Survey | SO2 and O3 measurements obtained from the European Climate Assessment and Dataset and the National Climatic Data Center | Quantitative | OLS, IV, SEM | Depression | Air quality was found to influence health outcomes. The Marginal Willingness to Pay (MWTP) for a one-unit reduction in SO2 and O3 were calculated at €221 and €88 annually, respectively. Using the Eurod mental health measure, these MWTP values were €155 and €68, respectively. | Yes, Yes | Social |
| Shin et al. [26], 2018 | Score 11/15. To examine the relationship between extended AP exposure and various mental health indicators, including subjective stress, depressive disorders, health-related QoL, and suicidal tendencies. | 124,205 adults aged 19+ living in their current domiciles for > five years | Korea | Survey | PM10, NO2, CO, sulfur dioxide data from Korean Air Pollutants Emission Service | Quantitative | Logistic regression | Depression, stress, suicide ideation | AP is linked to poor MH in men and young adults. Long-term exposure leads to higher stress, depression, and suicide ideation. | Yes, - | Emotional, Behavioral |
| Sui et al. [31], 2018 | Score 9/15. To conduct a cross-sectional investigation examining the potential relationship between AP exposure and MH outcomes. | 412 female college students | China | Questionnaire | PM2.5 data acquired from China's Environmental Protection Bureau | Quantitative | Linear regression | Depression, anxiety | No direct link between measured AP and MH due to estimation based on outdoor time, not actual levels. | No, - | Emotional, Behavioral, Social |
| Ren et al. [21], 2019 | Score 12/15. To examine how AP affects MH through cognitive and non-cognitive abilities. | 31,990 observations | China | Survey | PM2.5 by U.S. NASA | Quantitative | Structural equation modelling (SEM), Monte Carlo | Miscellaneous | Higher AP linked to worsened MH, partially mediated by non-cognitive and cognitive abilities, with stronger effects from PM2.5. | Yes, - | - |
| Roberts et al. [24], 2019 | Score 10/15. To address the association between air quality and MH problems. | 284 London twins born in 1994-1995 | England | Interviews, Self-reports | PM2.5, NO2 data from the London Atmospheric Emissions Inventory | Qualitative | Linear regression, Logistic regression | Depression | No associations between age-12 pollution exposure and concurrent MH problems. | No, - | Emotional |
| Xue et al. [20], 2019 | Score 9/15. To estimate associations between multiple environmental factors and self-assessed MH scores. | 17,447 Chinese adults aged 45+ | China | Survey | PM2.5 from Community Multiscale Air Quality Model | Quantitative | Statistics | Depression, nervousness, powerless | Significant association between higher levels of PM2.5 and MH scores decrease. | Yes, Yes | Emotional, Behavioral |
| Bernardini et al. [28], 2020 | Score 11/15. To demonstrate the relationship between daily levels of AP and daily admissions for MH disorders to hospitals. | 1,860 emergency department admissions | Italy | Electronic health records | PM10, PM2.5, O3, CO, NO2 data from the meteorological station of Perugia-Monteluce | Quantitative | Linear regression | Miscellaneous | Among the pollutants, only the O3 estimated coefficient is statistically significant at the 1% level. | No, - | Social |
| Gu et al. [32], 2020 | Score 10/15. To explore the relation between AP and MH. | 14,772 individuals | China | Survey | PM2.5 from NASA | Quantitative | Ordinary least squares (OLS), Two-stages least squares (2SLS), Interaction analysis | Depression, nervousness, powerless, restless or fidgety | Results found that AP significantly harms MH. PM2.5 increases the prominence of the four negative emotions.  Analysis confirms the link with women, rural residents, and low-income individuals at risk. | Yes, Yes | Emotional, Behavioral |
| Zhou et al. [29], 2020 | Score 10/15. To explore the association between AP and the MH and depression. | 150 counties | China | Survey | PM2.5, PM10, O3, SO2, NO2, CO from China National Environmental Monitoring Centre | Quantitative | Logistic regression | Depression | SO2 and CO exposure increases depression risk in elderly, especially affecting women, rural residents, and those with lower education/income. No firm evidence of an association. | No, - | Emotional, Behavioral |
| Ao et al. [62], 2021 | Score 11/15. To investigate AP impact for the same individuals over time on MH. | 28,962 observations of middle-aged and elderly participants | China | Survey, Questionnaire | PM2.5 calculated by retrospective analysis, NASA | Quantitative | Regression | Depression | AP associated with deteriorated MH in older adults, particularly among less educated individuals and women. Indoor and outdoor AP negatively impact elderly MH, substantially raising the risk of experiencing depressive symptoms. | Yes, Yes | Emotional |
| Bakolis et al. [33], 2021 | Score 11/15. To test the association of AP with MH. | 1,698 adults | England | Survey | PM data from the London Atmospheric Emissions Inventory | Quantitative | Linear regression, Logistic regression | Miscellaneous | Study finds higher AP exposure links to increased risk of mental disorders, physical symptoms, and psychotic experiences. Stronger effects seen in non-movers for NO2 and NOx exposure. | Yes, - | Behavioral, Physical |
| Hu et al. [35], 2021 | Score 11/15. To examine the impact of AP on MH. | 51 countries | - | Survey | PM2.5 from the World Bank (WD) database | Quantitative | Panel Tobit model, Mediation model, Bootstrap test | Depression, anxiety | Higher income is linked to less depression and anxiety. Poorer countries more impacted. Education and population density worsen effects. Bad physical health worsens MH with income levels. | Yes, Yes | Emotional, Behavioral |
| Kanner et al. [47], 2021 | Score 12/15. To evaluate the relationship between unspecified pregnancy-related mental disorders and depression with mean AP exposure during preconception, initial trimester, and full pregnancy period. | 234,022 pregnancies | USA | Electronic health records | PM2.5, PM10, CO, NO2, NOx, SO2, O3 data from Community Multiscale Air Quality models | Quantitative | Logistic regression | Depression, unspecified mental disorders | Pregnancy exposure to PM2.5, PM10, NO2, and NOx correlated with increased risk of mental disorders and depression.  CO exposure showed a decrease in risk. SO2 results were inconsistent. Exposure during preconception and first trimester also indicated links between pollutants and MH outcomes. | Yes, - | Emotional |
| Kim et al. [48], 2021 | Score 12/15. To investigate the long-term relationship between residential AP exposure and utilization of MH services among individuals with initial presentations of psychotic and mood disorders. | 38,101 participants, including 2,220 cancer survivors and 35,881 from the general population | Korea | Survey | PM10, NO2, SO2, CO data from nationwide monitoring stations by the Ministry of the Environment of Korea | Quantitative | Logistic regression, propensity score matching for AP and mental health links | Depression, stress, suicide ideation | PM10 impacts MH in cancer survivors. NO2, SO2, and CO do not affect MH. Air pollutants increase stress in the general population, except for SO2. | Yes, - | Emotional |
| Newbury et al. [46], 2021 | Score 14/15. To examine the longitudinal association between residential AP exposure and MH service use among individuals with first presentations of psychotic and MDs. | 82,000+ individuals | England | Electronic health records | NO2, Nox, PM2.5, PM10 data from London Atmospheric Emissions Inventory | Quantitative | Zero-inflated regression, Standard negative binomial regression | MDs, Psychosis | Exposure to residential AP was linked to increased use of MH services among individuals recently diagnosed with psychotic and mood disorders. These associations remained evident even after a 7-year period. | Yes, - | Behavioral |
| Petrowski et al. [34], 2021 | Score 10/15. To examine PM10's impact on MH and well-being factors (life satisfaction, stress resilience, anxiety, depression, self-esteem). | 3,020 adults | Germany | Questionnaire | PM10 from German Environment Agency | Quantitative | Linear regression | MH factors (stress resilience, life satisfaction, self-esteem, etc.) | Limited evidence of strong links between AP and MH determinants. | No, - | Emotional,  Physical |
| Yang et al. [37], 2021 | Score 12/15. To investigate the association between air pollutants and psychological well-being. | 52,568 individuals | China | Survey | PM2.5 data compiled by Washington University in St. Louis' Atmospheric Composition Analysis Group | Quantitative | Panel data regression | Depression | Higher AP linked to worse MH, especially in low-income individuals. Smoking worsens effects. Study emphasizes need to address AP's impact on MH with consideration of health behaviors. | Yes, - | Behavioral |
| Ahmed et al. [23], 2022 | Score 10/15. To examine the impact of PM2.5 and NO2 on emotional and behavioral aspects, as well as early developmental stages in children under 13 years old. | 5,471 children and mothers | Australia | Survey | PM2.5, NO2 data estimated from Satellite-based land-use regression models | Quantitative | Logistic regression | Emotional and Behavioral problems | Limited evidence suggested that exposure to PM2.5 during early life and childhood might be associated with emotional and behavioral problems, as well as delays in gross motor skill development. However, the majority of associations were found to be non-significant. | No, - | Emotional, Behavioral, Physical |
| Bruyneel et al. [40], 2022 | Score 12/15. To assess if short-term ambient pollutant fluctuations correlate with onset of MH-related work incapacity. | 12,270 observations | Belgium | Survey | NO2, O3, PM2.5, BC from Belgian Interregional Environment Agency | Quantitative | Logistic regression | Neurotic, stress-related, somatoform and MDs; life-management difficulty issues | NO2 had varying effects by age and season, with highest impact in the 40-49 age group and stronger associations in spring and summer. O3 effects differed based on the type of MH issue. | Yes, - | Behavioral, Physical |
| Hautekiet et al. [36], 2022 | Score 13/15. To evaluate the association between AP and self-rated MH. | 16,455 participants | Belgium | Questionnaire | NO2, PM2.5, and black carbon data obtained from Belgian monitoring stations and Corine Land Cover (CLC) database | Quantitative | Logistic regression | Depression, anxiety, stress, suicide ideation | Long-term exposure to PM2.5, black carbon, or NO2 was negatively associated with MH, with mediation by physical activity. | Yes, - | Emotional, Behavioral |
| Jiang and Chen et al. [38], 2022 | Score 12/15. To study the effects of AP on MH. | 337 Chinese cities | China | Survey | Air quality index from Ministry of Ecology and Environment of the People’s Republic of China | Quantitative | Fixed effects panel regression | Depression, episodic memory, cognitive ability | AP is linked to higher chance of severe mental illness, but foreign investment can lessen impact. Effects stronger in women, young people, less educated, and those in underdeveloped areas. | Yes, - | Social |
| Ju et al. [42], 2022 | Score 12/15. To evaluate the causal link between long-term AP exposure and the physical and psychological health of elderly individuals. | 7,042 participants aged 45+ | China | Survey | PM2.5 Hindcast Database | Quantitative | Correlated Random Effects Control Function | Depression, anxiety | PM2.5 associated with deteriorated physical and MH in middle-aged and older adults, elevating risks of chronic conditions, reduced physical well-being, depression, and anxiety. | Yes, Yes | Emotional, Behavioral, Physical |
| Sun et al. [39], 2022 | Score 14/15. To deduce the cause-effect relationship between levels of air pollutants and mental well-being. | 65,326 individuals | China | Survey, Questionnaire | PM10, NO2, SO2, CO, O3 data from the China Environment Yearbook | Quantitative | Fixed effects regression | Depression | PM10 negatively affects MH, particularly in urban areas and lower education levels. Chronic exposure to AP linked to MH problems. | Yes, Yes | Emotional, Behavioral |
| Balakrishnan et al. [49], 2023 | Score 13/15. To investigate the causal effects of AP on cognitive difficulties, feelings of inability to manage important life matters, and experiences of sadness. | 21,203 individuals aged 18+ | India | Survey | PM2.5 data obtained from the Central Pollution Control Board (CPCB) | Quantitative | Instrumental variable fixed effects model | Depression, stress | MH significantly worsened from AP exposure in the previous year. | Yes, Yes | Physical |
| Bhui et al. [44], 2023 | Score 9/15. To suggest future research priorities and strategies for addressing them. | - | - | - | - | Qualitative | Narrative review | Miscellaneous | Bad air is linked to MH issues. Kids and teens at higher risk. AP causes mental issues. | Yes, Yes | - |
| Cao et al. [50], 2023 | Score 11/15. To examine the hypothesis that increasing MH challenges among urban populations in developing nations may be linked to high levels of AP. | 252 cities | China | Search queries | Air quality index and specific pollutants (PM2.5) from from China’s Ministry of Ecology and Environment | Qualitative | Instrumental variable fixed effects model | Miscellaneous | AP harms urban MH more with longer exposure, impacting men and married individuals. Wealthy cities and green spaces have less impact. In China, a 1 SD rise in PM2.5 leads to 1.15 million affected. | Yes. Yes | Emotional, Behavioral |
| Ju et al. [43], 2023 | Score 12/15. To explore the causal effects of AP on MH and understand if physical activity could play a beneficial role. | 21,944 participants aged 18+ | China | Survey | PM2.5 from the Chinese Tracking Air Pollution | Quantitative | Instrumental variable fixed effects model | Depression | PM2.5 and ground ozone harm MH. Exercise helps, regardless of age, gender, income, or health, Regular exercise can protect MH from AP. | Yes, Yes | Emotional, Behavioral, Physical |
| Ronaldson et al. [45], 2023 | Score 12/15. To examine longitudinal associations between AP exposure and MH service use in people with dementia. | 5,024 elderly dementia patients | England | Electronic health records | PM2.5, NO2 data from the London Atmospheric Emissions Inventory | Quantitative | Negative binomial regression models | Depression, psychosis, dementia | Residential AP exposure is associated with increased CMHT usage among people with dementia. | Yes, - | Social |
| Wang et al. [9], 2023 | Score 14/15. To assess the combined impact of simultaneous exposure to various air pollutants and weather conditions on mental health outcomes. | 1.47M outpatient visits | China | Hospital data | SO2, CO, O3, NO2, PM10, and PM2.5 data obtained from the Qingyue Open Environmental Data Center | Quantitative | eGRU model analyzes AP and weather on MH SHAP technique shows their impact on risk | Depression, schizophrenia, anxiety, BD, ADHD, ASD | AP and weather worsen MH disorders. Nitrogen, sulfur, dioxide, humidity tied to depression, anxiety. Existing MH conditions susceptible to air pollutants and weather. | Yes, - | Emotional, Behavioral |
| Jáni et al. [30], 2024 | Score 10/15. To investigate the impact of AP and maternal depression on MH outcomes in young adulthood. | 202 mother–child dyads | Czech Republic | Questionnaire | PM, SO2, NOx | Quantitative | Linear regression | Depression, anxiety, MDs, psychosis | A correlation was found between NOX exposure patterns and maternal depression in early pregnancy. This relationship predicted mood regulation issues and schizotypal traits in young adults. | Yes, Yes | Emotional, Behavioural |
| Tota et al. [2], 2024 | Score 9/15. To provide a summary of the relationship between PM2.5, NO2, and SO2 exposure and the worsening of symptoms in depression, schizophrenia, and anxiety disorders. | - | - | - | - | Qualitative | Narrative review | Depression, anxiety, schizophrenia | PM2.5, NO2, and SO2 showed the strongest correlations with mental health disorders among the studied pollutants. | Yes, - | - |
| Lyons et al. [41], 2024 | Score 10/15. To investigate if AP may be associated with MH and wellbeing. | 8,504 community-dwelling adults, 4,674 observations | Ireland | Survey | PM2.5 | Quantitative | Logistic regression | Depression, anxiety | Evidence suggested associations between prolonged exposure to ambient PM2.5 and increased incidence of depression and anxiety. | Yes, - | Emotional, Social |
| Newbury et al. [25], 2024 | Score 11/15. To examine the longitudinal associations of AP exposure in pregnancy, childhood, and adolescence with psychotic experiences, depression, and anxiety | 9065 youths from ages 13 to 24 years | UK | Questionnaire | PM2.5, NO2, Noise pollution | Quantitative | Logistic regression | Depression, anxiety, psychosis | Pregnancy PM2.5 exposure was also associated with psychotic experiences and depression. | Yes, - | Emotional, Behavioural |
| Newbury et al. [60], 2024 | Score 11/15. To investigate the long-term relationships between AP exposure during pregnancy, childhood, and adolescence with psychotic experiences, depression, and anxiety. | 14,000 participants | UK | Survey | PM, Noise | Quantitative | Logistic regression | Depression, anxiety, psychosis | Exposure to PM2.5 during pregnancy was associated with increased occurrence of psychotic experiences and depression. | Yes, - | Emotional, Behavioural |

ADHD: Attention Deficit Hyperactivity Disorder; AP: Air Pollution; ASD: Autism Spectrum Disorder; BP: Bipolar Disorder; UV: Instrumental Variables; MD: Mood Disorder; MH: Mental Health; MWTP: Marginal Willingness-To-Pay; NASA: National Aeronautics and Space Administration; OLS: Ordinary Least Squares; PM: Particulate Matter; QoL: Quality of Life; SEM: Structural Equation Modelling.

**Table S2.** Chemical pollution and its effect on mental health (n=8).

| **Author** | **Study quality and aim** | **Sample size** | **Country** | **Personal Data** | **Pollution Data** | **Method** | **Analytic approach** | **Disorders** | **Findings/ Results** | **Association, Causality** | **Pollution outcomes** |
| --- | --- | --- | --- | --- | --- | --- | --- | --- | --- | --- | --- |
| Fluegge et al. [51], 2016 | Score 10/15. To investigate the association between PAH and child MH. | 600 mother-child pairs | USA | Survey | PAH | Quantitative | Linear regression | Miscellaneous | PAH exposure in pregnancy harms child MH at ages 7 and 11, while N₂O exposure has no impact. No interaction between the two - N₂O does not worsen the effects of PAH on child MH. | Yes, Yes | - |
| Ayuso-Álvarez et al. [52], 2019 | Score 12/15. To evaluate the association between soil concentrations of heavy metals and metalloids and the occurrence rates of mental health conditions. | 18,073 individuals in 1772 areas | Spain | Survey, Questionnaire | Concentrations of heavy metals and metalloids in surface soil, as documented in the Geochemical Atlas of Spain | Quantitative | Logistic regression | Emotional and Behavioral problems | High levels of Pb, As, Cd, and Mn in topsoil increase mental disorder risk, especially Pb and Cd. | Yes, - | - |
| Calloway et al. [53], 2020 | To qualitatively explore the PFAS exposure experience and associated stressors. | 9 participants | USA | Interviews | PFAS | Qualitative | Thematic analysis | Stress | PFAS contamination causes stress - health worries, uncertainty, distrust, financial strain. | Yes, - | Emotional, Behavioral, Social |
| Achatz et al. [55], 2021 | Score 9/15. To investigate the potential connection between long-term exposure to methylmercury (MeHg) and the deterioration of MH status. | 3 villages | Brazil | Interviews | Chronic methylmercury (MeHg) | Qualitative | Linear regression | Depression | The study revealed a trend towards poorer MH indicators among participants with higher levels of MeHg exposure. | Yes, No | Physical |
| Banwell et al. [54], 2021 | Score 9/15. To describe residents’ experiences and perceptions of PFAS. | 180 participants | Australia | Interviews | PFAS | Qualitative | Thematic analysis | Miscellaneous | Participants emphasized the anxiety and stress of dwelling in uncertainty regarding the possible socio-economic and health impacts of PFAS. | Yes, - | Emotional, Physical, Social |
| Schmitt et al. [12], 2021 | Score 10/15. To review existing studies and establish a foundation for future research on how contaminants of emerging concern (CEC) affect psychological well-being. | - | - | - | - | Qualitative, Quantitative | Systematic review | Miscellaneous | Meta-analysis: CEC has impact on MH. Exposure increases risk of issues.  Qualitative analysis: Risk factors include lack of trust, health concerns, limited resources. Review: Research limited; more investigation needed on CEC's impact on mental distress. | Yes, Yes | - |
| Legg et al. [13], 2023 | Score 10/15. To investigate the connection between living in environmentally contaminated areas and the mental health and psychological well-being of the residents. | - | - | - | - | Qualitative, Quantitative | Systematic review | Miscellaneous | The study found evidence supporting an association between MH issues and the experience of living in environmentally contaminated areas. | Yes, - | - |
| Tan et al. [56], 2023 | Score 12/15. To examine how exposure to polycyclic aromatic hydrocarbons (PAHs) affects the mental health of non-smokers, and how this impact may be influenced by alcohol consumption. | 1513 non-smoking employees | China | Survey, Questionnaire | Urinary OH-PAHs | Quantitative | Logistic regression model, generalized linear model | Miscellaneous | PAH exposure increases anxiety and depression. Alcohol consumption only leads to depression. High PAH and alcohol have a synergistic effect on depression, not anxiety. | Yes, Yes | Physical |

CEC: Chronic Environmental Contamination; MH: Mental Health; OH-PAH: Hydroxypolycyclic Aromatic Hydrocarbons; PAH: Polycyclic Aromatic Hydrocarbons; PFAS: Per- and Poly-Fluoroalkyl Substances.

**Table S3.** Noise pollution and its effect on mental health (n=5).

| **Author** | **Study quality and aim** | **Sample size** | **Country** | **Personal Data** | **Pollution Data** | **Method** | **Analytic approach** | **Disorders** | **Findings/ Results** | **Association, Causality** | **Pollution outcomes** |
| --- | --- | --- | --- | --- | --- | --- | --- | --- | --- | --- | --- |
| Hammersen et al. [57], 2012 | Score 11/15. To examine associations between NP and MH. | 19,294 adults | Germany | Survey | Noise from air traffic, neighbors, and road traffic | Quantitative | Bivariate analyses | Depression, anxiety, Behavioral/emotional control, general positive affect | High NP is linked to poor MH, except for air traffic. High NP doubles the risk of impaired MH. Road and neighbour noise also related to MH issues. Women more affected by NP than men. | Yes, - | Emotional, Behavioral |
| Ma et al. [58], 2018 | Score 11/15. To investigate NP potential effects on MH. | 1280 individuals | China | Survey, Questionnaire | Housing renovation/ construction noise, commercial noise, railway or subway noise | Quantitative | Bayesian multilevel logistic models | Miscellaneous | Higher NP exposure is significantly associated with worse MH. | Yes, - | Emotional, Behavioral |
| Zaman et al. [8], 2022 | Score 9/15. To review existing literature on the public health consequences and underlying biological mechanisms associated with ambient NP. | - | - | - | - | Qualitative | Systematic review | Miscellaneous | The study identified a relationship between exposure to NP and psychological and MH issues, including anxiety and depression. | Yes, - | - |
| Newbury et al. [25], 2024 | Score 11/15. To examine the longitudinal associations of NP exposure in pregnancy, childhood, and adolescence with psychotic experiences, depression, and anxiety. | 9065 youths from ages 13 to 24 years | UK | Questionnaire | Noise pollution | Quantitative | Logistic regression | Depression, anxiety, psychosis | Higher NP exposure in childhood and adolescence was associated with elevated odds for anxiety. | Yes, - | Emotional, Behavioural |
| Newbury Heron et al. [60], 2024 | Score 11/15. To investigate the relationship between nanoparticle (NP) exposure from gestation through age 12 and three specific mental health issues evaluated at ages 12, 18, and 24. | 14,000 participants | UK | Survey | PM, Noise | Quantitative | Logistic regression | Depression, anxiety, psychosis | Rare longitudinal evidence linking NP to anxiety. | No, - | Emotional, Behavioural |

MH: Mental Health; NP: Noise Pollution.

**Table S4.** Combined pollution and its effect on mental health (n=14).

| **Author** | **Study quality and aim** | **Sample size** | **Country** | **Personal Data** | **Pollution Data** | **Method** | **Analytic approach** | **Disorders** | **Findings/ Results** | **Association, Causality** | **Pollution outcomes** |
| --- | --- | --- | --- | --- | --- | --- | --- | --- | --- | --- | --- |
| Cuthbertson et al. [65], 2016 | Score 9/15. To present findings from a significant study on community mental health, specifically in the context of the Flint, Michigan water crisis. | Communities | USA | Survey with open questions | Water pollution | Qualitative | Grounded theory analysis | Miscellaneous | Flint water crisis is a major impact on residents' MH. Themes: stress, anxiety, depression, distrust of officials, fear of health effects, substance abuse as coping mechanism, ripple effects on Behavioral health. Low-income, African American communities hit harder. | Yes, - | Emotional, Behavioral |
| Attademo et al. [67], 2017 | Score 7/15. To summarize and synthesize work for EP and risk of psychotic disorders. | - | - | - | - | Qualitative | Narrative review | Schizophrenia | The study suggests that exposure to various environmental pollutants may be primary contributors to schizophrenia. These include xenobiotic heavy metals like lead and cadmium, components of AP such as PM, NOx, and SOx, as well as organic solvents and other elements of EP. | Yes, Yes | - |
| Dzhambov et al. [63], 2018 | Score 11/15. To investigate the links between exposure to air pollution (AP) and noise pollution (NP) in residential areas and the overall mental health of young adults, with emphasis on the mechanisms involved. | 720 students | Bulgaria | Questionnaire | Noise and NO2 | Quantitative | SEM | Depression, anxiety | There was an association between daily noise and MH. NO2 had no overall association with MH. | Air pollution: No, - Noise: Yes, - | Behavioral, Social |
| Lu et al. [68], 2018 | Score 9/15. To explore the health risk perception induced by smog. | 715 workers | China | Interviews, Questionnaire | Smog | Qualitative, Quantitative | Statistics | Miscellaneous | Skilled workers perceive more physical health risks from smog than MH risk (86.3%). | - | Behavioral |
| Klompmaker et al. [61], 2019 | Score 13/15. To examine relationships between combined exposure to air pollution (AP), noise pollution (NP), and surrounding green spaces with poor mental health. | 387,195 adults | Netherlands | Survey, Hospital data | PM and NO2 data by land-use regression models,  noise from the Standard Model Instrumentation for Noise Assessments | Quantitative | Logistic regression | Depression, anxiety, stress | In single exposures, AP was associated with poorer MH. Rail NP was associated with psychological distress. Road NP was associated with prescription of anxiolytics. In multi exposure analyses, relations with AP were attenuated. | Yes, - | Emotional |
| Thomson et al. [69], 2020 | Score 13/15. To examine an individual's view of their own well-being, assessing the relationship with various pollutants. | 398,300 respondents | Canada | Survey | PM2.5 by transport model, O3 and NO2 using Canadian AP Surveillance | Quantitative | Cox proportional hazard models | Miscellaneous | Long-term exposure linked to higher mortality risk. Poor mental and general health lead to greater mortality risk from pollutants, particularly NO2 and PM2.5. MH status likely influence susceptibility to AP mortality. | Yes, - | Emotional, Behavioral |
| Rodney et al. [1], 2021 | Score 9/15. To assess relation between MH and exposure to bushfire smoke. | 2,084 adults | Australia | Survey | Exposures to Bushfire and Smoke | Quantitative | Statistics | Depression, anxiety | 97% had smoke-related symptoms. Over half felt anxious/depressed, and women more affected. Pre-existing conditions, poor health, parenthood, direct fire impact led to worse health. Women had more MH issues. 17% sought medical advice, mainly from general practitioners. | Yes, - | Emotional, Behavioral, Physical, Social |
| Eisenman et al. [66], 2022 | Score 9/15. To perform a comprehensive scoping review of current literature examining the impact of wildfire smoke on well-being and MH. | - | - | - | - | Qualitative, Quantitative | Scoping review | Miscellaneous | Wildfire smoke damages MH. Limited evidence on specific effects. Studies indicate anxiety, depression, stress, PTSD, diminished life quality. Proposed model connects smoke exposure to MH problems. | Yes, - | - |
| Gignac et al. [71], 2022 | Score 10/15. To conduct a panel study to assess if AP can affect sleep quality, mood, attention, and perceived stress. | 2,135 teens | Spain | Questionnaire | PM2.5 NO2 and Noise | Quantitative | Linear regression | Stress | AP linked to externalizing for movers, noise causes externalizing issues. Social fragmentation is helpful for non-movers, deprivation and low social cohesion lead to internalizing problems. PM2.5 and noise related to internalizing but link unstable. Green space is not significant. | Yes, - | Emotional, Behavioral, Social |
| Wigand et al. [70], 2022 | Score 7/15. To analyze and visualize the global research landscape on mental health, climate change, pollution, and deforestation over 15 years. | - | - | - | - | Qualitative | Narrative review | Miscellaneous | Climate change, pollution and deforestation hurt global MH. | Yes, - | - |
| Hao et al. [64], 2022 | Score 11/15. To examine the cross-sectional associations of PM2.5 and road NP with MH disorders in men and women. | 500,000+ participants | China | Survey | PM2.5 and road traffic noise estimated with verified models | Quantitative | Logistic regression | Depression, nerves, anxiety, tension, BD | High PM2.5 is associated to more MH issues. Road noise affects MH too. Age, gender, and lack of sleep influence MH outcomes. | Yes, - | Emotional, Behavioral |
| Gómez González et al. [59], 2023 | Score 12/15. To investigate how environmental factors influence rates of hospital admissions. | 2191 observations with a total of 67,225 admissions | Spain | Hospital data | PM2.5, PM10, NO2, O3, and Noise | Quantitative | Linear regression | Miscellaneous | Admissions were significantly related in the short term with NP. No correlation between chemical AP and hospital admissions is observed. | Air pollution: No, - Noise: Yes, - | Social |
| Zeng et al. [72], 2023 | Score 12/15. To assess the long-term relationships between various environmental exposures and both externalized and internalized behavioral problems throughout the adolescent years. | 2,135 adolescents | Netherlands | Survey | PM2.5, Noise | Quantitative | Random-effect regression models | Depression, anxiety | Findings demonstrate that AP, NP are risk factors for adolescent MH. Exposure levels and exposure changes are of critical importance for adolescent MH. | Yes, - | Emotional, Behavioral |
| Radua et al. [81], 2024 | Score 12/15. To summarize and assess the credibility of the existing evidence on the impact of AP and climate change on MH outcomes through systematic reviews. | - | - | - | - | Quantitative | Umbrella review | Miscellaneous | This umbrella review confirms that AP and climate change represent intertwined elements of the main global crisis of our time. | Yes, - | - |

BD: Bipolar Disorder; EP: Environmental Pollution; NP: Noise Pollution; PM: Particulate Matter; SEM: Structural equation mode
